# The introduction of a mid-urethral stent for hypospadias surgery in toilet-trained children

**DOI:** 10.1007/s00383-024-05836-4

**Published:** 2024-09-23

**Authors:** Emmanuelle Seguier-Lipszyc, Andrew Shumaker, Kobi Stav, Anna Itshak, Amos Neheman

**Affiliations:** 1 Meir Medical Center, Tel Aviv University, Kfar Saba, Israel; 2 Assaf Harofeh Medical Center, Tel Aviv University, Tzrifin, Israel

**Keywords:** Hypospadias, Pediatric surgery, Urethroplasty, Stent, Continence, Toilet-trained

## Abstract

**Purpose:**

To address the unique challenges presented by hypospadias repair in toilet-trained boys, we propose a modification to the standard stenting technique: implementation of a mid-urethral stent (MUS) extending beyond the urethroplasty, terminating distally to the sphincter mechanism. This modification upholds continence while facilitating normal voiding.

**Methods:**

Toilet-trained boys undergoing hypospadias repair from 2009 to 2020 were retrospectively assessed. Patients were allocated into one of two groups: “Continent” drainage (a short stent was placed across the urethroplasty) or “incontinent” drainage (a standard stent or a Foley catheter was placed). Stent- related complications (dislodgement and obstruction) and surgical outcomes were compared.

**Results:**

545 children underwent hypospadias repair with 96 (17.6%) of them toilet-trained. The “continent” and “incontinent” groups consisted of 44 and 52 patients. No differences were found regarding age, severity of hypospadias, number of corrective procedures, operative time or surgical technique. Rates of stent-related complications did not differ. No significant difference was found regarding complications requiring additional surgery, including meatal stenosis and dehiscence. Post-operative fistula occurred in one patient in the continent group and in seven patients in the incontinent group.

**Conclusion:**

Use of a continence-preserving MUS is a safe alternative in toilet-trained patients undergoing hypospadias repair without increasing risk of complications.

## Introduction

Hypospadias is a common congenital disorder of the male genitalia with an estimated prevalence of 34.2/10000 births [1]. Surgical repair is usually performed prior to the initiation of “potty training”, most often in the first year of life.

Toilet-trained hypospadias patients undergoing primary or repeat surgery represent a small but unique subgroup. Regression to a state of urinary incontinence as a result of post-surgical urethral stenting necessitates the use of diapers or an indwelling catheter. Such drainage can potentially impair ambulation, limit the child’s autonomy and have a negative psychologic impact [2].

Continent drainage in this subset of patients can easily be achieved by shortening the urethral stent and positioning of the distal end to dwell distal to the membranous urethra.

In this study, our aim is to evaluate the safety and feasibility of continent drainage utilization in toilet-trained boys undergoing hypospadias repair. We hypothesize that this simple modification will not adversely affect outcomes.

## Material and methods

A retrospective review of our prospectively maintained database of patients with hypospadias at our institution between the years 2009 and 2020 was performed. Ethical review board approval for the study was obtained (IRB-0064-17-ASF). We included all toilet-trained boys scheduled for primary or secondary distal hypospadias surgery (fistula, meatal stenosis or dehiscence repair). We excluded all patients with proximal hypospadias who underwent a two-stage repair with buccal or preputial grafting. All cases were performed by a single surgeon.

**Fig. 1:**
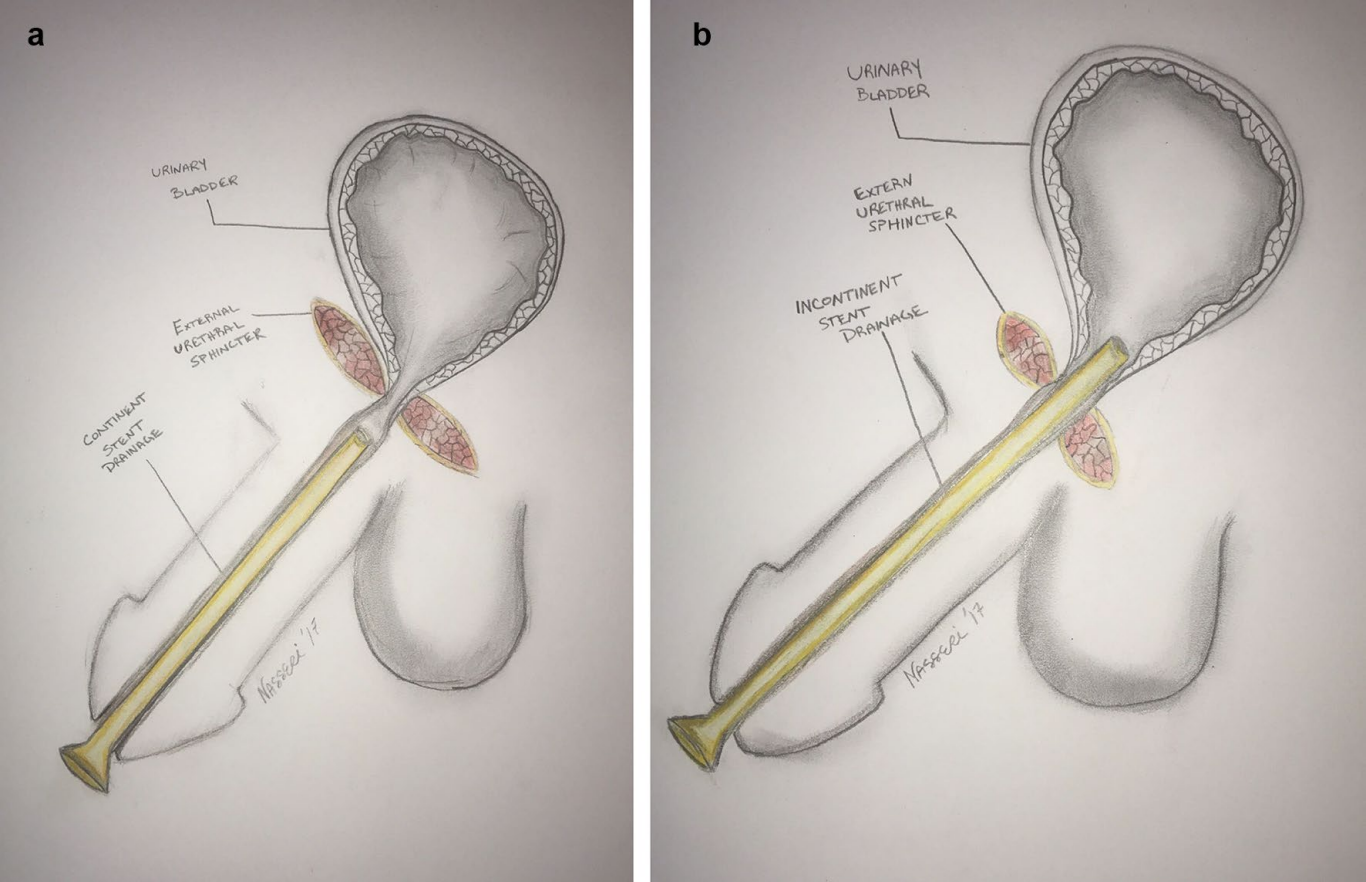
**a** Continent stent positioning. Note placement distal to external urethral sphincter. **b** Incontinent stent positioning. Note placement of stent proximal to external urethral sphincter and into bladder

Patients were randomized and allocated to one of two groups. The “continent” drainage group received post-surgical urethral stent placement distal to the membranous urethra to maintain continence (Fig. 1a). The “incontinent” drainage group received either a urethral stent reaching the bladder and draining to a diaper (Fig. 1b) or a Foley catheter with a leg bag or catheter plug placed.

An 8 Fr Zaontz stent (Cook Medical, Bloomington, IN, USA) was used in the “continent” group. The stent was initially inserted into the bladder and then retracted gradually until urine ceased to flow. At this point, it could be surmised that the tip of the stent was located distal to the external sphincter. The stent was then removed, shortened accordingly, reinserted and secured to the glans with a 5-0 prolene stay suture (Fig. 1a).

In both groups the same dressing was used.

Follow-up visits were scheduled for stent removal one week post-operatively, and subsequent assessments were conducted at 3, 6, and 12 months after the surgery to evaluate outcomes. Data regarding these outcomes were retrieved from the institutional medical records.

The study outcomes included: (1) stent-related complications such as urinary retention or stent dislodgement, and (2) surgery-related complications including meatal stenosis, fistula, and dehiscence.

Complications were defined as follows. Meatal stenosis was diagnosed based on parental reporting of thin or deflected urinary stream combined with a stenotic appearing meatus observed during physical examination at follow-up. Glans dehiscence was defined according to the criteria outlined by Snodgrass and Bush, which involves complete glans wing separation resulting in a subcoronal meatus [3]. Fistula was diagnosed if urine extravasation was observed from any opening other than the meatus during physical examination.

All statistical analysis was performed using SPSS statistics^©^ v.25 (IBM, Armonk, NY, USA). Continuous variables were described with medians and interquartile range (IQR). Due to lack of normal distribution, study group and sub-group analyses were performed using the Mann–Whitney *U* test which calculated the *p* values in the tables. Categorical variables were described with frequencies and percentages and compared using the Chi-square test of independence. Statistical significance was set at *p* < 0.05.

## Results

A total of 545 patients were included based on the study parameters, 96 of these patients (17.6%) were toilet-trained at the time of surgery. Patient demographic and pre-surgical data are presented in Table 1. Overall, no significant differences were found between the study groups with regard to patient age (*p* = 0.31), severity of hypospadias prior to surgery (*p* = 0.25), surgical indication (*p* = 0.91) or rate of primary vs. secondary surgery (*p* = 0.39). Surgical data are presented in Table 2. Between the two groups, there were no significant differences in the proportions of penile-straightening procedures, median operative times or surgical technique employed.

**Table 1:** Pre-surgical data of “continent” and “incontinent” groups

Variable	“Continent” (*n* = 44)	“Incontinent” (*n* = 52)	*p *value
Median age at operation (months, IQR)	63.0 (46.5–111.5)	48.5 (34–81.5)	0.31
Operation (%)			
Primary	23 (52.3)	29 (55.8)	0.39
Secondary	21 (47.7)	23 (44.2)	
Indication for secondary surgery (%)			
Fistula	11 (27.3)	11 (21.2)	0.91
Meatal stenosis	9 (20.5)	11 (23.1)	
Chordee	1 (2.3)	1 (1.9)

**Table 2:** Surgical data of “continent” and “incontinent” groups

Variable	“Continent” (*n* = 44)	“Incontinent” (*n* = 52)	All patients (*n* = 96)	*p* value
Penile-straightening procedure (%)	8 (18.2)	17 (34)	25 (26)	0.08
Surgical technique (%)				
TIP	9 (20.4)	18 (34.6)	27 (28.1)	0.27
MAGPI	12 (27.3)	6 (11.5)	18 (18.7)	
Fistula repair	12 (27.3)	13 (25)	25 (26)	
Meatoplasty	9 (20.4)	13 (25)	22 (22.9)	
Urethral advancement	2 (4.5)	2 (3.8)	4 (4.2)	
Median operative time (min, IQR)	83.5 (45.2–98.3)	66 (47.0–98.0)	74 (47–98)	0.71
Median duration of stent dwelling (days, IQR)	6 (5–8)	8 (7–8)	7 (5.5–8)	0.06

Post-operative outcomes are presented in Table 3. Median follow up for the entire cohort was 13.9 months (IQR 4.6–18.9). In the “continent” stent group, 4 patients (9.1%) had a stent-related complication. Two patients had dislodgement of the stent, two other patients developed urinary retention (due to post-operative pain and compression of the stent and to stent obstruction). In all stent-related complications, the stent was replaced with an 8 Fr silicone catheter and no further complications were observed. In the “incontinent” group, 2 cases (3.8%) of catheter obstruction were diagnosed.

**Table 3:** Summary of post-operative outcomes

Complication	“Continent” (*n* = 44)	“Incontinent” (*n* = 52)	All patients (*n* = 96)	*p* value
Meatal stenosis (%)	6 (13.6)	4 (7.7)	10 (10.4)	0.34
Fistula formation (%)	1 (2.3)	7 (13.5)	8 (8.3)	**0.04**
Dehiscence (%)	0	1 (1.9)	1 (1)	0.35
Stent-related complication (%)	4 (9.1)	2 (3.8)	5 (5.2)	0.37
Additional surgery required (%)	7 (15.9)	10 (19.2)	17 (17.7)	0.67

Statistically significant values are in bold (*p* < 0.05)

Regarding complications not stent related, the only statistically significant difference was that the “incontinent” group presented more post-operative fistulas.

## Discussion

The use of a stent in hypospadias surgery aims to reduce both short and long-term post-operative complications; meatal stenosis, urethral fistula, and dehiscence being the principal issues that develop in 10% of distal and 33% of proximal primary repairs [4]. Various alternative methods of urine drainage after surgery have been described [1, 5, 6]. While some authors describe omission of this stent in hypospadias repair, the majority of surgeons opt to use various forms of drainage to bypass the urethroplasty.

Buson et al. compared stented and non-stented patients undergoing meatal-based flap urethroplasty. A higher complication rate was observed in the non-stented group, with urinary retention developing in 19% of patients and urethrocutaneous fistula in 14% compared to 0–6%, respectively, in the stented group [7]. Another study stated that distal hypospadias patients could undergo surgery with no stent in toilet-trained children, but confirmed there were lower post-operative complications when a stent was used [8]. The use of a continent mid-urethral stent (MUS) was previously described. Mitchell et al. first reported the use of a urethral “splent” (silicone pleated stent) in 1986. None of the patients in this series required subsequent catheterization or hospitalization and urethral fistula developed in 4.5% of patients [9]. In our series urethral fistula developed in 13.5% in the “incontinent” group and only in 2.3% in the “continent” group.

Toilet-trained boys scheduled for hypospadias repair, both primary and secondary, represent a unique subgroup of hypospadias patients. Surgery alone has been proven a significant source of emotional trauma for the pediatric population [10]. While often only being for a limited period of time, the loss of continence experienced by children may be traumatic and may lead to discomfort in the post-operative period. Several studies have demonstrated the detrimental effects of long-standing incontinence with regard to quality of life and self-esteem. Children with nocturnal enuresis report lower self-esteem scores compared to continent children and show an improvement in self-esteem following treatment for this issue [11]. Gladh et al. observed that incontinent children 6–8 years of age suffer the greatest discrepancy in self-reported quality-of-life scores compared to continent children [12]. To the best of our knowledge, no existing literature has assessed the psychologic effects of temporary continence loss, especially due to iatrogenic causes, on quality of life.

An additional concern for the parents of boys with incontinent drainage following hypospadias surgery is the management of a catheter and leg bag or the subjecting their child to the discomfort of a diaper. Ambulation becomes cumbersome and the child’s active daily living can become restricted. Our method of continent drainage solves this issue. All patients in the “continent” drainage group were discharged on post-operative day 0 or 1 following repair, and 40/44 (91%) patients required no further management during their recovery period.

Although we did not report the rate of bladder spasms nor that of post-operative urinary tract infection in this study, it has been well-documented in other studies. Bladder spasm is commonly seen in toilet-trained boys following hypospadias repair. A meta-analysis performed by Mousavi et al. suggested that up to 30% of toilet-trained boys undergoing hypospadias repair complained of bladder spasm [13]. In a recent prospective, randomized trial, El-Karamany et al. reported a 47% rate of bladder spasms in toilet-trained boys who underwent hypospadias repair with a stent [14]. A continent MUS has potential to reduce symptoms aggravated by indwelling bladder drainage. We believe that further investigation is required to assess the efficacy of continent drainage in reducing bladder symptoms.

This cohort represents a relatively older group undergoing hypospadias repair. Furthermore, more than half (61%) of the patients in the “continent” stent group, and roughly half (52%) of patients overall were re-do cases of hypospadias repair. We believe that this may have resulted in a cohort at increased risk for complications in comparison to younger children undergoing primary repair. Kocherov et al. reported on their experience with 84 toilet-trained boys who underwent hypospadias repair with an overall complication rate of 27% [15]. Garnier et al. reported complications for patients undergoing primary hypospadias repair and noted that those over the age of two years at the time of surgery were at significantly higher risk of developing post-operative complications (OR 1.98, *p* = 0.002) [16]. Our study reported an overall complication rate of 25% and 17.7% requiring additional intervention. Regarding secondary repair and complication risk, Snodgrass et al. analyzed 1536 patients and reported that each prior attempted repair resulted in a 1.5-fold increase in complication risk (OR 1.51, CI 1.25–1.83) [4]. These studies describe findings which coincide with our own experience in a similar age group and reflect the complicated nature of hypospadias repair in older children.

At our institution, the use of MUS has become the standard of care for toilet-trained boys undergoing hypospadias repair. This modification holds potential advantages, not only for toilet-trained boys but also for non-toilet-trained boys undergoing the procedure. Furthermore, well designed studies focusing on younger non-continent boys are needed to explore the potential benefits of MUS in preventing bladder spasms and reducing post-operative complications such as urinary bladder infections. If proven beneficial, the widespread adoption of MUS in hypospadias repair could significantly improve outcomes for a majority of patients. However, the true benefit of this modification is yet to be studied and defined.

Our study is not without limitations. Data regarding pain, bladder spasms, and need for anticholinergic medication after surgery were not collected in this study. We did not address quality of life, ambulation limitations, or importantly, any regressive behavior that may have occurred after stent or catheter removal. These are significant questions to be answered and should be reported in future studies. We do believe, however, that in an appropriately selected patient, continent drainage offers advantages. For patients in whom there is little concern that bladder access may be lost, continent drainage may be utilized without additional risk of complications.

## Conclusion

Continent preserving MUS is a safe and simple option in toilet-trained patients undergoing hypospadias repair without an increased risk of complications.

## Data Availability

No datasets were generated or analyzed during the current study.

## Abbreviations

IQR: Interquartile range
TIP: Tubularized incised plate urethroplasty
MAGPI: Meatal advancement and glanduloplasty
MUS: Mid-urethral stent
